# ﻿Two new *Trichoderma* species (Hypocreales, Hypocreaceae) isolated from decaying tubers of *Gastrodiaelate*

**DOI:** 10.3897/mycokeys.99.109404

**Published:** 2023-09-08

**Authors:** Chuwen Ye, Tingting Jing, Yuru Sha, Minghe Mo, Zefen Yu

**Affiliations:** 1 Laboratory for Conservation and Utilization of Bio-resources, Key Laboratory for Microbial Resources of the Ministry of Education, Yunnan University, Kunming, Yunnan, 650091, China Yunnan University Kunming China

**Keywords:** Multi-gene phylogeny, plant disease, taxonomy, Trichoderma

## Abstract

Species of *Trichoderma* are widely distributed around the world. In this study, two new species in *Trichoderma*, named as *T.albidum* and *T.variegatum*, were introduced and illustrated. These species were isolated from diseased tubers of *Gastrodiaelata* in China and identified based on morphological characteristics and multi-gene sequence analyses of three loci that is the internal transcribed spacer regions of the ribosomal DNA (ITS), the translation elongation factor 1-α encoding gene (*tef1-α*) and the gene encoding the second largest nuclear RNA polymerase subunit (*rpb2*). Distinctions between the new species and their close relatives were discussed. According to results of the phylogenetic analyses, *T.albidum* belonged to the Harzianum clade and *T.variegatum* are grouped with species of the Spirale clade. The expansion of two clades provided research foundations for the prevention and control of tuber diseases in *G.elata*.

## ﻿Introduction

*Trichoderma* Pers. is important ecologically and economically. These fungi are widely used in agriculture, industry and medicine, including being used as bio-fungicides to control plant diseases, and as regulators of plant growth, fortifiers of soil fertility, and producers of antibiotics and enzymes (Lorito et al. 2010; Saravanakumar and Kathiresan 2014; Bischof et al. 2016; Adnan et al. 2017; Zhang et al. 2022). Furthermore, some species have great potential to remediate soil and water pollution as well as to manufacture gold or silver nanoparticles (Harman et al. 2004; Anand et al. 2006; Mazyar et al. 2010). However, several species were reported as the causal agents of green mold disease in mushroom cultivation, the disease of *Gastrodiaelata* Bl. 1856 and opportunistic pathogens of humans (Park et al. 2006; Komon-Zelazowska et al. 2007; Sandoval-Denis et al. 2014; Han et al. 2017).

*Trichoderma* is a hyper-diverse fungal genus. Members of *Trichoderma* are widely distributed in a variety of ecosystems, including natural soils, decaying wood and bark, and living plant tissues (Samuels et al. 2006; Samuels et al. 2012; Jaklitsch and Voglmayr 2015; Zhang and Zhuang 2017). The initial species-level identification of the genus was based on their morphological characteristics. Nevertheless, as more species were discovered, taxonomic studies of the genus became increasingly complicated due to overlapping morphological traits among species (Druzhinina et al. 2005; Han et al. 2017; Barrera et al. 2021; Zhang et al. 2022). Misidentification species can have profound negative impacts on plant quarantine, industrial applications, and health of human and animal (Sandoval-Denis et al. 2014; Chaverri et al. 2015).

With the development of the times and the progress of science and technology, our research on fungal phylogeny has gradually transitioned from relying on morphological methods to relying on molecular biology methods. DNA sequence analysis was introduced and has provided more reliable identification for *Trichoderma* species. Numerous loci were considered for use in *Trichoderma* identifications and phylogenetic analyses, e.g. internal transcribed spacer regions of the ribosomal DNA, the translation elongation factor 1-α encoding gene, the gene encoding the second largest nuclear RNA polymerase subunit, α-actin, calmodulin, chitinase 18-5 (Kullnig-Gradinger et al. 2002; Druzhinina et al. 2012; Chaverri et al. 2015; Chen and Zhuang 2017a; Zhang et al. 2022). Specifically, *tef1-α* and *rpb2* have facilitated rapid and accurate species identifications and have been used in the phylogenetic analyses and identification of novel species of *Trichoderma* in recent years (Cai et al. 2022). Analyses using only ITS may only be able to identify to the genus level or lead to errors due to fragment length, copy number and other problems, so it is necessary to add rpb2 and tef1 to improve the systematic analysis. It was shown that the multi-gene sequence analysis of ITS, rpb2 and tef1 could identify 60% of the current *Trichoderma* species, while the other loci were not suitable for gene barcoding due to the small gene size and distribution range (Cai et al. 2022). In contrast, *cal* and *chi1*8-5 are rarely used due to their missing adequate sequence data or low sequence variability (Druzhinina et al. 2012; Bissett et al. 2015; Jaklitsch and Voglmayr 2015; Zhu and Zhuang 2015; Qin and Zhuang 2016). Furthermore, the large subunit of ATP citrate lyase (*acl*1) was recently introduced for taxonomic research of the genus, which turns out to be efficient (Jaklitsch et al. 2013). Currently, the combination of multi-loci phylogenetic analyses and phenotypic characteristics have been extensively used for species delineation of *Trichoderma*. Relying on this method, a number of species which were misclassified previously have been re-identified as new species, so the number of species in the genus has increased dramatically. (Plessis et al. 2018; Innocenti et al. 2019). In addition, morphological approaches remain important to validate and complement the phylogenetic results.

*Trichoderma* contains more than 400 species belonging to different clades and Harzianum clade and Sprale clade are two of them (Wijayawardene et al. 2020). Since the systematic revision of species in the Harzianum clade was provided by Chaverri et al. (2015), a large number of new species have been described and recorded. The Harzianum clade now contains more than 60 species (Gu et al. 2020). Green ascospores are a common feature of the Harzianum clade (Zhu and Zhuang 2015). Species in Harzianum clade have antifungal properties and bio-control ability, and they can effectively suppress soil-borne plant pathogens. Most of the species can be isolated from soil, rotting wood, other fungi, and plant endophytes (Chaverri et al. 2015). *Trichodermaharzianum* is one of the most well-known species in Harzianum clade. The Spirale clade is smaller in size compared to the Harzianum clade. The Spirale clade was identified as a separate terminal branch by Jaklitsch and Voglmayr (2015), and was authenticated by later researchers (Chen and Zhuang 2017a). *T.hunanense* K. Chen & W.Y. Zhuang, *T.longisporum* K. Chen & W.Y. Zhuang, and *T.spirale* Bissett are the species of this clade (Chen and Zhuang 2017a). Species in Spirale clade share the following similar features: producing yellow pigments on plates, possessing oblong conidia and forming hairy pustules (Chen and Zhuang 2017a). Identification and complementation of species in two clade is of significance to enrich the species diversity of both branches.

In the present study, 78 isolates obtained from the diseased *Gastrodiaelata* Blume collected from Xiaocaoba, Zhaotong were found to belong to *Trichoderma* after preliminary identification and classification by ITS sequence. Based on morphological characteristics and DNA sequence data at three loci: the genes encoding RNA polymerase II subunit (*rbp*2) and translation elongation factor 1-α gene (*tef1-α*), and ITS regions of the nuclear ribosomal RNA gene, a new species belonging to the Harzianum clade and the other belonging to the Spirale clade were described and illustrated.

## ﻿Materials and methods

### ﻿Sample collection and isolation

Tubers of *Gastrodiaelata* with rot symptoms were collected from Xiaocaoba, Yiliang County, Zhaotong city, Yunnan province, China. Samples were placed in sterile plastic bags, labeled, and transported to the laboratory. Infected *G.elata* were first washed in running tap water and autoclaved water, then surface disinfection with consecutive immersions was conducted for 30 s in 75% ethanol, 2 min in 1.5% sodium hypochlorite, then they were finally rinsed three times with autoclaved water and air-dried. Symptomatic tissues were cut into about 5 × 5 mm slices and placed on potato dextrose agar (PDA; 200 g potato, 20 g dextrose, 18 g agar, 1000 ml distilled water) plates. Petri dishes were sealed, incubated at 25 °C, and examined periodically. A small amount of hyphal tip cells was picked up and transferred to PDA medium when fungi grew out from infected tissues. The pure strains were further transferred and incubated on PDA, cornmeal agar (CMA; 20 g cornmeal, 18 g agar, 1000 ml distilled water) and synthetic low nutrient agar (SNA; 1 g KH_2_PO4, 1 g KNO_3_, 0.5 g MgSO_4_, 0.5 g KCl, 0.2 g glucose, 0.2 g sucrose, 18 g agar, 1000 ml distilled water) at 25 °C. After incubation, the colony and the microscopic morphology on PDA, CMA and SNA plates were observed, measured and photographed. Microscopic observations were performed using a BX51 microscope (Olympus) and with sterile water as a mounting medium for microscopy. Microscopic structures such as mycelium, conidiophores, conidia and phialides were observed and photographed, and at least 30 individuals of data were measured for each structure. Colony colors (surface and reverse) were confirmed based on Rayner’s color charts (Rayner 1970).

The pure cultures and dried cultures were deposited in the Herbarium of the Laboratory for Conservation and Utilization of Bio-resources, Yunnan University, Kunming, Yunnan, P. R. China ( YMF).

### ﻿DNA extraction, amplification and sequencing

DNA was extracted from fresh mycelia harvested from PDA plates after 4 days of incubation at 25 °C. 0.5g fungal mycelia we collected was transferred into a 1.5ml microcentrifuge tube with 0.7–0.8ml lysis buffer (7 mol/L Urea, 50 mmol/L Tris-HCl, 62.5 mmol/L NaCl, 1% SDS). The mixture was spun at 12000 r/min for 5 min and the aqueous phase was transferred into a new 1.5 ml tube. An equal volume of DNA extract (phenol/chloroform/ isoamyl alcohol, 25:24:1) was added into the homogenates. The mixture was spun at 12000 r/min for 5 min and the aqueous phase was transferred into a new 1.5 ml tube. The homogenates containing DNA were re-extracted by adding an equal volume of isopropanol and 1/10 volume of 3 mol/L NaAc. The mixture was placed at -20 °C for 20 min and then centrifuged at 12000 r/min for 5 min, and the aqueous phase was discarded. The DNA pellet was washed with 70% ethanol twice in order to precipitate them, dried, and re-suspended in 50 μl H_2_O for PCR (Sun et al. 2000; Liu et al. 2005). Fragments of the internal transcribed spacers (ITS), RNA Polymerase II subunit B (*rpb2*), and translation elongation factor 1-alpha (*tef1-α*) were amplified with the three primer pairs: ITS4 and ITS5 for ITS (White et al. 1990), f*rpb2*-5f and f*rpb2*-7cr for *rpb2* (Liu et al. 1999), and EF1-728F (Carbone and Kohn 1999) and TEF1LLErev (Jaklitsch et al. 2005) for *tef1-α*, respectively. A 25 μl reaction volume contained 1.0 μl DNA template, 1.0 μl of each forward and reverse primers, 12.5 μl 2× MasterMix (Tiangen Biotech) and 9.5 μl dd H_2_O. The PCR thermal cycle programs of the amplification followed Chaverri (Chaverri et al. 2011) and Chen (Chen and Zhuang 2017a). PCR products were purified with the PCR product purification kit (Biocolor BioScience and Technology Co., Shanghai, China), and forward and reverse sequencing was carried out on an ABI 3730 XL DNA sequencer (Applied Biosystems, Foster City, California) with primers used during PCR amplification. The sequences were deposited in the GenBank database at the National Center for Biotechnology Information (NCBI) and the accession numbers were listed in Table 1.

**Table 1.** Strains and the GenBank accession numbers analyzed in this study.

Species	Strain	GenBank accession number		
		ITS	RPB	TEF
*Protocreafarinosa*	CBS 121551	MH863119	EU703935	EU703889
*Protocreapallida*	CBS 299.78	MH861137	EU703948	EU703900
*T.achlamydosporum*	YMF 1.06226*	MN977791	MT052180	MT070156
*T.afarasin*	DIS 314F	FJ442259	FJ442778	FJ463400
*T.afroharzianum*	CBS 124620*	FJ442265	FJ442691	FJ463301
*T.afroharzianum*	GJS 04-193	FJ442233	FJ442709	FJ463298
*T.aggregatum*	HMAS 248863*	KY687946	KY688001	KY688062
*T.aggregatum*	HMAS 248864	KY687947	KY688002	KY688063
*T.aggressivum*	CBS 100525	AF057600	AF545541	AF348095
*T.aggressivum*	DAOM 222156*	AF456924	FJ442752	AF348098
*T.alni*	CBS 120633*	EU518651	EU498349	EU498312
*T.alni*	CPK 2494	EU518652	EU498350	EU498313
*T.alpinum*	HMAS 248821*	KY687906	KY687958	KY688012
*T.alpinum*	HMAS 248830	KY687912	KY687961	KY688015
*T.anaharzianum*	YMF 1.00241	MH262584	MH262577	MH236493
*T.anaharzianum*	YMF 1.00383*	MH113931	MH158995	MH183182
*T.asiaticum*	YMF 1.00168	MH262582	MH262575	MH236492
*T.asiaticum*	YMF 1.00352*	MH113930	MH158994	MH183183
*T.azevedoi*	CEN 1422*	MK714902	MK696821	MK696660
*T.azevedoi*	CEN 1423	MK714903	MK696822	MK696661
*T.bannaense*	HMAS 248840*	KY687923	KY687979	KY688037
*T.bannaense*	HMAS 248865	KY687948	KY688003	KY688038
*T.breve*	HMAS 248844*	KY687927	KY687983	KY688045
*T.breve*	HMAS 248845	KY687928	KY687984	KY688046
*T.brunneoviride*	CBS 120928	EU518661	EU498358	EU498318
*T.brunneoviride*	CBS 121130*	EU518659	EU498357	EU498316
*T.camerunense*	CBS 137272*	AY027780	NA	AF348107
*T.camerunense*	GJS 99-231	AY027783	NA	AF348108
*T.ceraceum*	GJS 95-159	AF275332	AF545508	AY937437
*T.cerinum*	DAOM 230012*	KC171336	KJ842184	KJ871242
*T.christiani*	CBS 132572*	NA	KJ665244	KJ665439
*T.christiani*	S93	NA	KJ665245	KJ665442
*T.concentricum*	HMAS 248833*	KY687915	KY687971	KY688027
*T.concentricum*	HMAS 248858	KY687941	KY687997	KY688028
*T.dacrymycellum*	WU 29044	FJ860749	FJ860533	FJ860633
*T.epimyces*	CBS 120534*	EU518663	EU498360	EU498320
*T.epimyces*	CPK 2487	EU518665	EU498361	EU498322
*T.guizhouense*	HGUP 0038*	JN191311	JQ901400	JN215484
*T.guizhouense*	S628	NA	KJ665273	KJ665511
*T.hainanense*	HMAS 248837*	KY687920	KY687976	KY688033
*T.hainanense*	HMAS 248866	KY687949	KY688004	KY688034
*T.harzianum*	CBS 226.95*	AJ222720	AF545549	AF348101
*T.harzianum*	GJS 05-107	FJ442679	FJ442708	FJ463329
*T.helicolixii*	CBS 133499*	NA	KJ665278	KJ665517
*T.helicolixii*	CBS 135583	NA	KJ665277	KJ665516
*T.hengshanicum*	HMAS 248852*	KY687935	KY687991	KY688054
*T.hengshanicum*	HMAS 248853	KY687936	KY687992	KY688055
*T.hirsutum*	HMAS 248834*	KY687916	KY687972	KY688029
*T.hirsutum*	HMAS 248859	KY687942	KY687998	KY688030
*T.hunanense*	HMAS 248841*	NR_154571	KY687980	KY688039
*T.hunanense*	HMAS 248867	KY687950	KY688005	KY688040
*T.ingratum*	HMAS 248822*	KY687917	KY687973	KY688018
*T.ingratum*	HMAS 248827	KY687909	KY687966	KY688021
*T.italicum*	CBS 132567*	NA	KJ665282	KJ665525
*T.italicum*	S15	NA	KJ665283	KJ665526
*T.koreanum*	SFC20130926-S008	NA	MH025989	MH025983
*T.koreanum*	SFC20131005-S066*	MH050352	MH025988	MH025979
*T.lentinulae*	CGMCC 3.19848	MN594470	MN605868	MN605879
*T.lentinulae*	HMAS 248256*	MN594469	MN605867	MN605878
*T.liberatum*	HMAS 248831*	KY687913	KY687969	KY688025
*T.liberatum*	HMAS 248832	KY687927	KY687970	KY688026
*T.linzhiense*	HMAS 248846*	KY687929	KY687985	KY688047
*T.linzhiense*	HMAS 248874	KY687957	KY688011	KY688048
*T.longisporum*	HMAS 248843*	KY687926	KY687982	KY688043
*T.longisporum*	HMAS 248868	KY687951	KY688006	KY688044
*T.neotropicale*	CBS 130633*	MH865818	NA	HQ022771
*T.parepimyces*	CBS 122768	FJ860801	FJ860563	FJ860665
*T.parepimyces*	CBS 122769*	MH863234	FJ860562	FJ860664
*T.peberdyi*	CEN1425	MK714905	MK696824	MK696663
*T.peberdyi*	CEN1426*	MK714906	MK696825	MK696664
*T.pinicola*	KACC 48486 *	MH050354	MH025993	MH025981
*T.pinicola*	SFC20130926-S014	NA	MH025991	MH025978
*T.pleuroti*	CBS 124387*	HM142363	HM142372	HM142382
*T.pleuroti*	CPK 2117	NA	NA	EU279975
*T.pleuroticola*	CBS 124383*	HM142362	HM142371	HM142381
*T.pleuroticola*	TRS70*	KP009264	KP009172	KP008951
*T.polypori*	HMAS 248855*	KY687938	KY687994	KY688058
*T.polypori*	HMAS 248861	KY687944	KY688000	KY688059
*T.propepolypori*	YMF 1.06199	MN977790	MT052182	MT070157
*T.propepolypori*	YMF 1.06224*	MN977789	MT052181	MT070158
*T.pseudodensum*	HMAS 248828*	KY687910	KY687967	KY688023
*T.pseudodensum*	HMAS 248829	KY687911	KY687968	KY688024
*T.rifaii*	CBS 130746*	FJ442663	NA	FJ463324
*T.rifaii*	DIS 337F	FJ442621	FJ442720	FJ463321
*T.rufobrunneum*	HMAS 266614*	KF729998	KF730010	KF729989
*T.rufobrunneum*	isolate 8155	NA	KF730007	KF729992
*T.rugulosum*	SFC20180301-001*	MH050353	MH025986	MH025984
*T.rugulosum*	SFC20180301-002	NA	MH025987	MH025985
*T.simile*	YMF 1.06201*	MN977793	MT052184	MT070154
*T.simile*	YMF 1.06202	MN977794	MT052185	MT070153
*T.simplex*	HMAS 248842*	KY687925	KY687981	KY688041
*T.simplex*	HMAS 248860	KY687943	KY687999	KY688042
*T.solum*	HMAS 248847	KY687930	KY687986	KY688049
*T.solum*	HMAS 248848*	KY687931	KY687987	KY688050
*T.spirale*	DIS 173A	FJ442217	FJ442705	FJ463371
*T.spirale*	E425	NA	MK044189	MK044096
*T.spirale*	E510	NA	MK044198	MK044105
*T.stramineum*	CBS 114248*	AY737765	AY391945	AY737746
*T.stramineum*	TAMA 0425	AB856609	AB856748	AB856675
*T.subazureum*	YMF 1.6185	MN977799	MT052190	MT070148
*T.subuliforme*	YMF 1.6182	MN977796	MT052187	MT070151
*T.subuliforme*	YMF 1.6183	MN977797	MT052188	MT070150
*T.subuliforme*	YMF 1.6184	MN977798	MT052189	MT070149
*T.vermifimicola*	CGMCC 3.19850	MN594472	MN605870	MN605881
*T.vermifimicola*	HMAS 248255*	MN594473	MN605871	MN605882
*T.xixiacum*	CGMCC 3.19698	MN594477	MN605875	MN605886
*T.xixiacum*	HMAS 248253*	MN594476	MN605874	MN605885
*T.zayuense*	HMAS 248835*	KY687918	KY687974	KY688031
*T.zayuense*	HMAS 248836	KY687919	KY687975	KY688032
*T.zelobreve*	CGMCC 3.19696	MN594475	MN605873	MN605884
*T.zelobreve*	HMAS 248254*	MN594474	MN605872	MN605883
*T.albidum*	YMF 1.7530*	OQ517962	OQ559127	OQ559118
*T.albidum*	YMF 1.7531	OQ517963	OQ559128	OQ559119
*T.variegatum*	YMF 1.7532	OQ517964	OQ559129	OQ559120
*T.variegatum*	YMF 1.7533*	OQ517965	NA	OQ559121
*T.variegatum*	YMF 1.7534	OQ517966	OQ559130	OQ559122

Notes: NA, not applicable. *, type strains.

### ﻿Phylogenetic analyses

Sequences of ITS, *rbp*2, and *tef1-α* of 111 strains, representing 59 species with close phylogenetic relation to two new species based on blast result of ITS sequence were downloaded from GenBank. Among them, 98 strains belong to the Harzianum clade and 11 strains belong to the Spirale clade, with *Protocreafarinosa* Berk. & Broome (CBS 121551) and *P.pallida* Ellis & Everh. Jaklitsch et al. (CBS 299.78) as the outgroups. Both the reference sequences and newly generated sequences in this study were listed in Table 1. DNA sequence data of each locus were aligned, respectively, by Clustalx 1.83 (Thompson et al. 1997) with the default parameters. Aligned sequences of multiple loci were manually adjusted and concatenated using BioEdit v.7.0 (Hall 1999). Finally, we obtained the combined sequence matrix (Fasta file) generated by BioEdit v.7.0, containing 2530 characters from three genes (522 from ITS, 833 from *rpb2*, 1178 from *tef1-α*).

Maximum Likelihood (ML) and Bayesian inference (BI) analyses were conducted to allocate the phylogenetic positions of the new species. Maximum Likelihood analysis was computed by RAxML (Stamatakis 2006) with the PHY files generated with ClustalX 1.83 (Thompson et al. 1997), using the GTR-GAMMA model. Maximum likelihood bootstrap proportions (MLBP) were computed with 1000 replicates. Under the best fit model, Bayesian Inference (BI) analysis was performed with MrBayes v3.1.2 (Huelsenbeck and Ronquist 2001) with the NEXUS file converted by MEGA7 (Kumar et al. 2016). The best fit evolutionary model for each dataset was determined using MrModeltest 2.3 and incorporated into the analyses. A Markov Chain Monte Carlo (MCMC) algorithm of four chains was started in parallel from a random tree topology with the heating parameter set to 0.3. The MCMC analysis was run until the average standard deviation of the split frequencies dropped below 0.01 with trees saved each 1,000 generations. The initial 25% of the generations of MCMC sampling were discarded as the “burn-in” and posterior probabilities determined from the remaining trees. The Tree was viewed in FigTree v1.4 (Rambaut 2012), values of Maximum likelihood bootstrap proportions (MLBP) greater than 70% and Bayesian inference posterior probabilities (BIPP) greater than 85% at the nodes are shown along branches.

## ﻿Results

### ﻿Phylogenetic analyses

Phylogenetic positions of the new species were determined by analyses of the combined *tef1*, *rpb2* and ITS dataset containing 2533 characters. In our analyses, the 116 strains included 100 strains belonging to the Harzianum Clade, 14 strains belonging to the Spirale Clade and two outgroup taxa.

**Figure 1.**
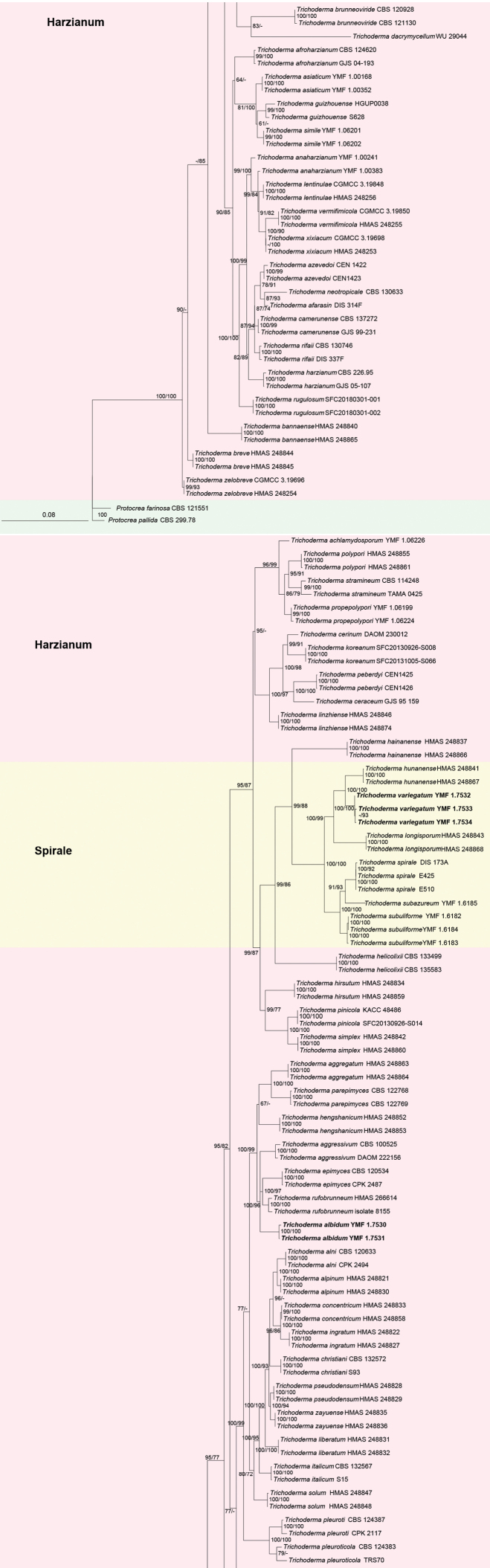
Phylogenetic tree of *Trichoderma* species based on the combined ITS, *tef1-α* and *rpb2* gene sequences constructed using Maximum-likelihood (ML) analysis and Bayesian inference (BI) analysis. The former values near nodes represent Bayesian posterior probabilities over 85% and the latter represent bootstrap support from ML bootstrap support over 70%. *Protocreafarinose* CBS 121551 and *P.pallida* CBS 299.78 were used as outgroups. Bold font indicates newly described species.

The ML analysis showed similar tree topology and was congruent with that obtained in the BI analysis (Fig. 1). The tree topology showed that our strains belonged to two new species, one new species classified in the Harzianum clade and the other one in the Spirale clade. Two strains were grouped together in an independent clade in the Harzianum clade and associated with *T.epimyces* Jaklitsch, *T.rufobrunneum* Z.X. Zhu & W.Y. Zhuang and *T.aggressivum* Samuels & W. Gams, designated as *T.albidum* (BIPP/MLBP = 100%/100%). In the Spirale clade, our three isolates formed one corresponding to a new species, designated as *T.variegatum* (BIPP/MLBP = 100%/100%).

### ﻿Taxonomy

#### ﻿ Trichoderma albidum

Taxon classificationFungiHypocrealesHypocreaceae

Z.F. Yu & T.T. Jing sp. nov.

7A7D17A9-A849-565C-86B2-049292370FB2

MycoBank No: 847677

Fig. 2

##### Etymology.

Referring to the rare white, whitish colonies on cultures media.

##### Description.

***Sexual morph***: Unknown. ***Asexual morph***: Conidiophores straight or slightly curved, branches mostly asymmetrically arranged, also paired, sometimes at irregular intervals along the main axis, closely-spaced, often orientated toward the apex, rarely forming secondary branches. Phialides lageniform to somewhat subulate, straight or slightly curved, often with a narrow neck, often in whorls of 2-5, (6.0–) 8.3–13.5 (–15.7) × (2.9–) 3.2–4.6 (–4.8) µm, l/w ratio (1.5–) 2.0–4.0 (–4.5), (1.5–) 1.9–3.2 (–3.8) µm wide at the base, widest around the middle. Conidia ovoid to subglobose, sometimes oblong, hyaline, smooth, (3.5–) 3.7–5.3 (–6.3) × (3.2–) 3.3–4.2 (–4.8) µm (mean = 4.4 × 3.8 μm, n=50), l/w ratio 1.0–1.3 (–1.8).

##### Culture characteristics.

Optimum temperature for growth 25 °C. No growth at 35 °C in CMA, PDA and SNA.

Colony radius on CMA after 3 days: 9–11 mm at 25 °C, 5–6 mm after 6 days at 30 °C, covering the plate after 11 days at 25 °C. Colony white to whitish, radial, not zonate, aerial hyphae sparse, arachnoid. Conidiation start after 4 days. Chlamydospores rare. No distinct odor noted, no diffusing pigment observed.

Colony radius on PDA after 3 days: 22 mm at 25 °C, 11 mm at 30 °C, covering the plate after 7 days at 25 °C. Colony dense, pale white, finely zonate, circular, aerial hyphae abundant, fluffy. Conidiation start after 8 days, formed numerously on aerial hyphae. No distinct odor noted, no diffusing pigment observed.

Colony radius on SNA after 72 h: 14 mm at 25 °C, 4 mm at 30 °C, covering the plate after 8 days at 25 °C. Colony hyaline, indistinctly zonate, aerial hyphae scarcely degenerating. Conidiation start after 7 days. Chlamydospores rare. No distinct odor noted, no diffusing pigment observed.

##### Materials examined.

China. Yunnan province, Zhaotong city, Yiliang county, Xiaocaoba Town, on diseased *Gastrodiaelata*, 25 Oct. 2021, T.T. Jing (holotype YMF 1.7530). Ibid. (culture: YMF 1.7531).

##### Notes.

Phylogenetically, *T.albidum* is associated with *T.aggressivum*. In comparison, *T.aggressivum* grows faster on PDA (50.5–56.0 mm after 3 days at 25 °C) and SNA (58.5–62.2 mm after 3 days at 25 °C), and produces shorter and narrower phialides ((4.0–) 5.7–7.8 (–21.0) × (1.3–) 2.7–3.5 (–4.3) µm) and much smaller green conidia (3.2–3.3 × 2.8–2.9 μm) (Samuels et al. 2002).

**Figure 2.**
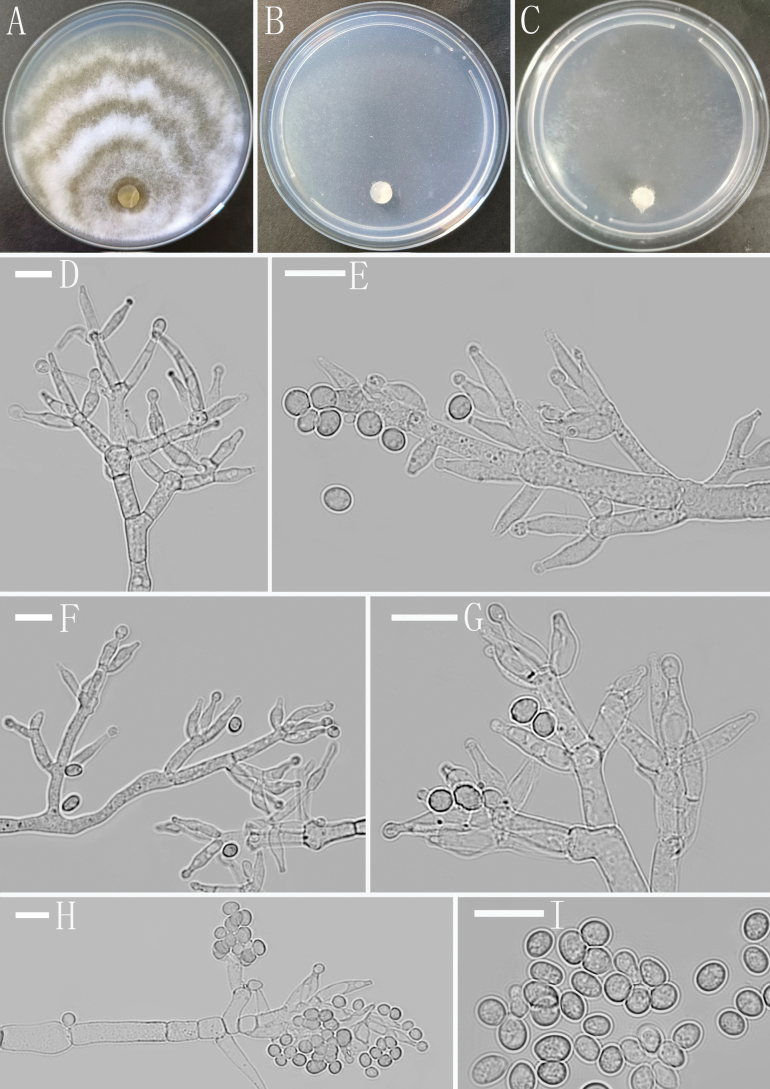
Morphology of *Trichodermaalbidum* (YMF 1.7530) **A–C** cultures on PDA, 7d; SNA, 8d; CMA, 11d **D–H** conidiophores and phialides **I** conidia. Scale bars: 10 μm (**D–I**).

#### ﻿ Trichoderma variegatum

Taxon classificationFungiHypocrealesHypocreaceae

Z.F. Yu & T.T. Jing sp. nov.

F1CD298B-69C1-5F15-9309-9AFB0AAF3824

MycoBank No: 847676

Fig. 3

##### Etymology.

Referring to the luteous, orange to amber, variable coloration of the colonies on cultures media.

##### Description.

***Sexual morph***: Unknown. ***Asexual morph***: Conidiophores straight or curved, asymmetry, sparsely branches, cylindrical mostly sterile to the apex. Often with a main axis, frequently tips sterile, disposed, relatively distant distribution at right angles to the axis or slightly oriented towards the conidiophore terminus, often solitary, not or rebranched once. Phialide lageniform to subulate, sometimes cylindrical, often with a narrow neck, discrete or integrated, solitary or in whorls of 2–3 (–4), (9.5–) 9.8–14.6 (–15.4) × (3.2–) 3.5–5.4 (–6.7) µm, l/w ratio (1.9–) 2.1–4.3 (–4.6), 2.2–3.4 µm wide at the base, widest around the middle. Conidia ellipsoidal to oblong, sometimes obovate, green, smooth, (4.3–) 4.7–7.4 (–8.6) × (2.7–) 3.0–4.1 (–4.3) µm (mean = 6.1 × 3.5 μm, n=50), l/w ratio (1.0–) 1.4–2.0 (–2.2).

##### Culture characteristics.

Optimum temperature for growth 25 °C. No growth at 35 °C in CMA, PDA and SNA.

Colony radius on CMA after 72 h: 20–22 mm at 25 °C, 14–15 mm at 30 °C, covering the plate after 8 days at 25 °C. Colony hyaline, indistinctly zonate, aerial hyphae nearly lacking. Conidiation starting after 8 days, formed on aerial hyphae. Chlamydospores common, subglobose to globose, smooth, terminal and intercalary, 5.3–13.4 × 5.0–11.3 µm. No distinct odor noted, yellow pigment noted.

Colony radius on PDA after 72 h: 30–32 mm at 25 °C, 27–29 mm at 30 °C, cover the plate after 6 days at 25 °C. Colony dense, aerial hyphae abundant, margin slightly lobed, forming numerous small yellow pigment droplets on the surface in the mature phase. Conidiation started after 8 days, formed on aerial hyphae. Chlamydospores abundant, subglobose to globose, smooth, terminal and intercalary, 5.5–10.6 × 5.3–10.1 µm. No distinct odor noted, yellow to brownish pigment diffusing into the agar.

Colony radius on SNA after 72 h: 18–22 mm at 25 °C, 17–19 mm at 30 °C, covering the plate after 8 days at 25 °C. Colony hyaline, not zonate, aerial hyphae sparse, relatively abundant at margin, arachnoid. Conidiation formed after 7 days, formed on aerial hyphae. Chlamydospores common, subglobose to globose, smooth, terminal and intercalary, 5.8–12.3 × 5.7–11.4 µm. No distinct odor noted, yellow pigment noted.

##### Materials examined.

China. Yunnan province, Zhaotong city, Yiliang county, Xiaocaoba Town, on diseased *Gastrodiaelata*, 25 Oct 2021, T.T. Jing (holotype YMF 1.7533). Ibid. (cultures: YMF 1.7532 and YMF 1.7534).

##### Notes.

Phylogenetically, *T.variegatum* is closely related to *T.hunanense* in the Spirale clade. *T.hunanense* can be easily distinguished by much shorter conidia ((3.6–) 4.2–5.6 (–7.5) µm) and not producing chlamydospore (Chen and Zhuang 2017a). Moreover, *T.hunanense* grows faster on PDA (46–47 mm after 3 days at 25 °C) and SNA (27–28 mm after 3 days at 25 °C) and forms green pustules in culture compared with *T.variegatum* (Chen and Zhuang 2017a).

**Figure 3.**
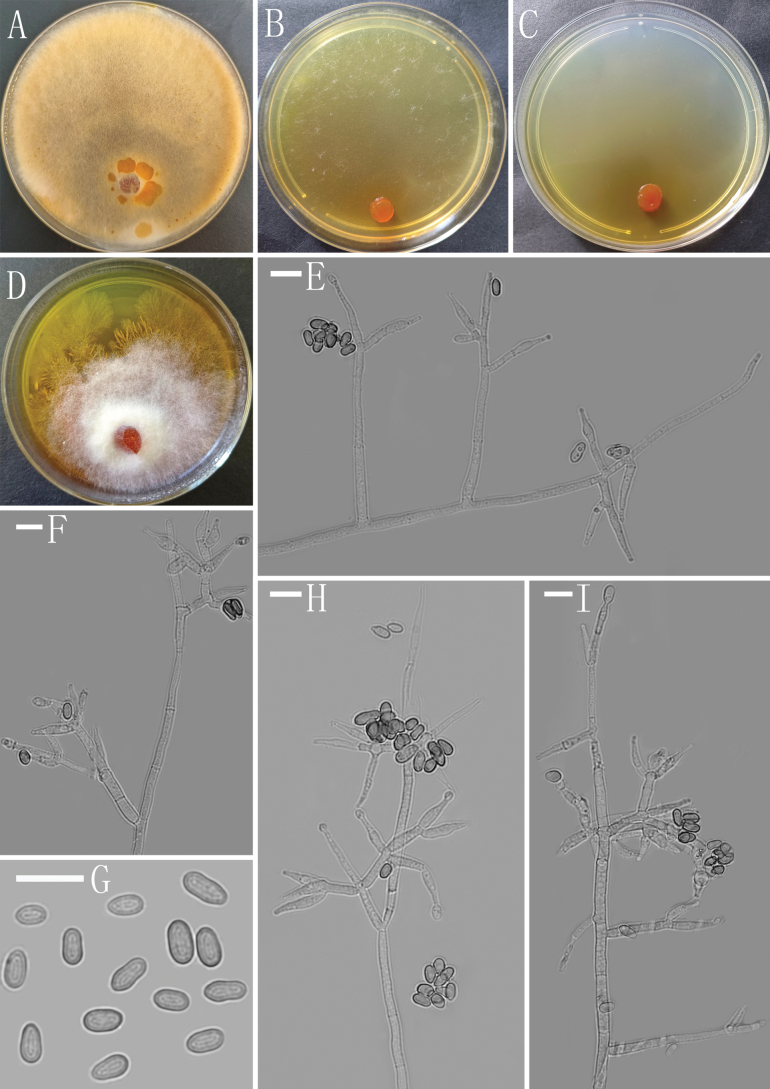
Morphology of *Trichodermavariegatum* (YMF 1.7533) **A–D** cultures on PDA, 8d; SNA, 9d; CMA, 9d; PDA, 4d **E, F, H, I** conidiophores and phialides **G** conidia. Scale bars: 10 μm (**E–I**).

## ﻿Discussion

At present, the combination of phylogenetic, morphological, ecological, and biogeographic data has effectively resolved all the known species within the genus *Trichoderma*. Specifically, two genes, *rpb2* and *tef1-α*, are widely deployed for identifications of new *Trichoderma* species. The present study employed a multilocus phylogenetic analysis for three molecular markers (ITS, *rpb2* and *tef1-α*) and morphological comparisons to delimit and recognize species within two clades of *Trichoderma*. Our analyses showed that our two novel *Trichoderma* species belonged to the Spirale clade and the Harzianum clade.

New specie, *T.variegatum*, is described here as a member of the Spirale clade, which is newly introduced by Chen and Zhuang to accommodate three *Trichoderma* species, *T.hunanense*, *T.longisporum* and *T.spirale* (Chen and Zhuang 2017a). *T.spirale* was first described by Bissett (Bissett 1991). However, the phylogenetic position of *T.spirale* was variable in the initial analyses. Chaverri and Samuels reported that *T.spirale* was closest to *T.polysporum* Rifai in the Polysporum clade (Chaverri and Samuels 2003). Subsequently, *T.spirale* was placed into the Strictipile clade by Jaklitsch and was closely related to *T.longipile* Bissett and *T.strictipile* Bissett (Jaklitsch 2009). Whereas, *T.spirale* was considered as a separate terminal branch in Jaklitsch and Voglmayr’s study (Jaklitsch and Voglmayr 2015). Afterwards, Chen and Zhuang introduced the Spirale clade to accommodate three aforementioned *Trichoderma* species in 2017 (Chen and Zhuang 2017a). Zheng et al. later added two species, *T.subuliforme* and *T.subazureum*, into the Spirale clade (Zheng et al. 2021). Members of the Spirale clade generally form hairy pustules, produce yellow pigments in culture and have more or less oblong conidia (Chen and Zhuang 2017a). *T.variegatum* is morphologically different from others in the Spirale clade in that it does not form hairy pustules in culture. Previously reported species of the Spirale clade were all isolated as saprobes from soil (Zhang and Zhuang 2017; Zheng et al. 2021). It is the first time that species of the clade have been found in plant tissues, confirming that species in the Spirale clade have flexible nutrition modes and potentially novel diversity in plants.

*T.albidum* belongs to the Harzianum clade, which is a cosmopolitan and ubiquitous group. The *T.harzianum* species complex is well known for its antifungal properties and effective bio-control capacity, often applied to restrain soil-borne plant pathogens (Chaverri et al. 2015; Degenkolb et al. 2015; Bunbury-Blanchette and Walker 2019; Gu et al. 2020). The Harzianum clade displays a complicated speciation history and heterogeneous morphology (Atanasova et al. 2010; Druzhinina et al. 2010; Qin and Zhuang 2017). Members of the Harzianum clade generally exhibit great variation in the number and size of pustules formed in culture, type of conidiophores and shape of phialides and conidia (Chaverri and Samuels 2003; Jaklitsch 2009; Qin and Zhuang 2017; Zheng et al. 2021). The taxonomy of species in the Harzianum clade was revised and the identity of commercial strains of *T.harzianum* was performed by Chaverri et al. in 2015 (Chaverri et al. 2015). Since then, multitudes of new species of the Harzianum clade have been reported and more than 70 species have been placed in the clade (Jaklitsch and Voglmayr 2015; Chen and Zhuang 2017a, b; Qiao et al. 2018; Zhang and Zhuang 2018; Phookamsak et al. 2019; Gu et al. 2020; Inglis et al. 2020; Barrera et al. 2021; Bustamante et al. 2021; Nuangmek et al. 2021). No doubt many species of this clade remain to be discovered.

The habitat of *Trichoderma* is highly heterogeneous, including agricultural fields, prairies, forests, salt marshes, and even desert (Gond et al. 2007; Verma et al. 2007; Gazis and Chaverri 2010). Some taxa of *Trichoderma* are contributing in suppressing or attacking other plant pathogens through their secondary metabolites, which have been explored as potential biological control agents (Degenkolb et al. 2008; Cardoso Lopes et al. 2012; Cheng et al. 2012; Degenkolb et al. 2015; Zhu et al. 2017; Bunbury-Blanchette and Walker 2019). On the contrary, a few species, such as *T.atrobrunneum* F.B. Rocha et al., *T.pleuroti* and *T.pleuroticola* S.H. Yu & M.S. Park were considered as causal agents of “Green mold” disease of the cultivated mushroom *Agaricusbisporus* (J.E. Lange) Imbach (Sandoval-Denis et al. 2014; Innocenti et al. 2019). *T.aggressivum*, which is closely related to *T.albidum*, also was reported to cause enormous damages to mushroom production (Oda et al. 2009; Schuster and Schmoll 2010; Kim et al. 2012; Kim et al. 2013). In our survey, we isolated and identified the fungi from diseased *G.elata* tissues, and obtained more than 250 isolates. 78 isolates were determined to be *Trichoderma* after preliminary identification by ITS barcoding. At present, the potential effects of these fungi on *G.elata* cultivation remain largely unknown. However, they likely represent a (yet to be confirmed) but growing number of fungal pathogens capable of infecting crop plants (Xu 2022). This study sets the foundation for future pathogenicity and epidemiological studies of these three *Trichoderma* species on *G.elata*, contributing to the future prevention and controls of tuber diseases in *G.elata* crop fields

## Supplementary Material

XML Treatment for Trichoderma albidum

XML Treatment for Trichoderma variegatum

## ﻿Additional information

### Data availability

All of the data that support the findings of this study are available in the main text. All sequences have been deposited in GenBank at the accession numbers given in the text.
